# An Improved Method of Handling Missing Values in the Analysis of Sample Entropy for Continuous Monitoring of Physiological Signals

**DOI:** 10.3390/e21030274

**Published:** 2019-03-12

**Authors:** Xinzheng Dong, Chang Chen, Qingshan Geng, Zhixin Cao, Xiaoyan Chen, Jinxiang Lin, Yu Jin, Zhaozhi Zhang, Yan Shi, Xiaohua Douglas Zhang

**Affiliations:** 1 School of Software Engineering, South China University of Technology, Guangzhou 510006, China; xinzhengdong@163.com; 2 Zhuhai Laboratory of Key Laboratory of Symbolic Computation and Knowledge Engineering of Ministry of Education, Zhuhai College of Jilin University, Zhuhai 519041, China; 3 Faculty of Health Sciences, University of Macau, Taipa, Macau 999078, China; yb67646@connect.umac.mo (C.C.); yb67647@connect.umac.mo (Y.J.); 4 Guangdong General Hospital, Guangdong Academy of Medical Science, Guangzhou 510080, China; gengqs2010@163.com; 5 Beijing Engineering Research Center of Diagnosis and Treatment of Respiratory and Critical Care Medicine, Beijing Chaoyang Hospital, Beijing 100043, China; 18301564184@163.com (Z.C.); shiyan@buaa.edu.cn (Y.S.); 6 Department of Endocrinology, First Affiliated Hospital of Guangzhou Medical University, Guangzhou 510120, China; gzscxy@126.com (X.C.); 13794353925@163.com (J.L.); 7 School of Law, Washington University, St. Louis, MO 63130, USA; zhazhang59@gmail.com; 8 Department of Mechanical and Electronic Engineering, Beihang University, Beijing 100191, China; 9 BARDS, Merck Research Laboratories, Upper Gwynedd, PA 19454, USA

**Keywords:** sample entropy, missing values, physiological data, complexity, medical information

## Abstract

Medical devices generate huge amounts of continuous time series data. However, missing values commonly found in these data can prevent us from directly using analytic methods such as sample entropy to reveal the information contained in these data. To minimize the influence of missing points on the calculation of sample entropy, we propose a new method to handle missing values in continuous time series data. We use both experimental and simulated datasets to compare the performance (in percentage error) of our proposed method with three currently used methods: skipping the missing values, linear interpolation, and bootstrapping. Unlike the methods that involve modifying the input data, our method modifies the calculation process. This keeps the data unchanged which is less intrusive to the structure of the data. The results demonstrate that our method has a consistent lower average percentage error than other three commonly used methods in multiple common physiological signals. For missing values in common physiological signal type, different data size and generating mechanism, our method can more accurately extract the information contained in continuously monitored data than traditional methods. So it may serve as an effective tool for handling missing values and may have broad utility in analyzing sample entropy for common physiological signals. This could help develop new tools for disease diagnosis and evaluation of treatment effects.

## 1. Introduction

The demand for more advanced, more personalized treatments, increased availability of healthcare and an aging population are pushing the market and expanding medical device technology, especially in the area of wearables for continuous monitoring of physiological signals. These advancements require better analytic methods to extract the useful information contained in these data more accurately due to the huge amount of data generated by these devices. Entropy, an indicator for the degree of irregularity in a dynamic system, has been applied to more and more disciplines since its definition was extended to information theory in 1950s [1]. As a nonlinear dynamic index, sample entropy (one type of entropy) is often used to measure the complexity of the physiological system in medical research for disease diagnosis and prognosis. Besides the routine detected indicators, this index can help doctors better confirm the diagnosis and prognosis, so as to provide better treatment and suggestions for patients’ rehabilitation. One of the most well-known examples is the use of entropy as an indicator of heart rate variability to evaluate cardiac sympathetic and parasympathetic functions [2]. In addition to the widely used application in the diagnosis and prognosis of the cardiac autonomic nerve disease [3,4], entropy is also used in the diagnosis of diabetes [5,6,7,8,9], chronic obstructive pulmonary disease [10,11,12] and other diseases [13]. The change of entropy values (decrease or increase) have been shown to be a predictor of multiple diseases [3,10,11].

The data from signals measured by continuous monitoring devices, such as wearables [7,14], commonly have various degrees of missing values [15] because of the patient’s unconscious movement, loose equipment and interference by other equipment. This issue of missing data is compounded by a study [16] which showed that sample entropy can be highly sensitive to missing values. Once the data has missing fragments, entropy fluctuations will be large. More worrisome, the abnormal fluctuations will increase as the percentage of missing values increases [17]. Handling missing values in the calculation of entropy is therefore imperative. Although there are a few studies to investigate the effect of missing values on the analytic results of nonlinearity including entropy [16,17], methods to deal with missing values have yet to be developed for the calculation of entropy. 

Currently, there are two basic strategies to overcome this problem: ignoring/imputing missing values and modifying the method of calculating sample entropy [18]. The issue is that common methods for imputing missing values may not work effectively for entropy calculation [19]. An artificial effect will be introduced to the calculated value of entropy [15] if missing values cannot be imputed in the same way as the distribution of the original data. In this paper, we propose a new method to improve the algorithm of calculating sample entropy on data with missing values that does not involve imputing missing values before entropy calculation. This provides a less intrusive way of handling missing values in the analysis of sample entropy for continuously monitoring physiological data since the new method does not add new data points. Thus removing the danger that the original structure of the data is compromised. 

To demonstrate the utility of our proposed method, the following key questions need to be answered: Can the new method be applied to common types of continuously monitoring physiological data? Is the new method robust to the data size represented by the length of a time series? Is it robust to the scheme of generating missing values? How does the new method perform compared with existing methods? In this paper, we designed simulations based on experimental data to address these questions. 

Our article is organized as follows: in the Methods section, we first introduce the definition of sample entropy and four methods of handling missing values. Then we present the datasets and the method we used to construct a sequence with missing values from the original sequence without missing values. In the Results section, we investigate the utility and applicability of our method to most common physiological signal types as well as the robustness of our method to both data size and scheme for generating missing values, as compared to the currently, commonly used methods. We conclude with discussion on the applicability and robustness of our method.

## 2. Methods

### 2.1. Sample Entropy

Sample entropy is a measure of irregularity, which was first proposed by Richman and Moorman [20,21]. It is defined as the negative natural logarithm of the conditional probability that two sequences similar for *m* points remain similar at the next point with a tolerance *r*.

Let $X_{i}=\left\{x_{1},\ldots ,x_{i},\ldots ,x_{N}\right\}$ represent a time series of length *N*. We define the template vector of length *m* from *X*: $X_{m}\left(i\right)=\left\{x_{i},x_{i+1},x_{i+2},\ldots ,x_{i+m-1}\right\}$ and the distance function between two such vectors:

(1) $$d\left(m,i,j\right)=d\left[X_{m}\left(i\right),X_{m}\left(j\right)\right]=\underset{k}{\max}\left\{\left|x_{i+k-1}-x_{j+k-1}\right|\right\},\;\mathrm{where}\;k=1,\ldots ,m$$

Then given a distance threshold *r*, the number of (similar or matched) vectors that are within *r* of the distance between the vectors $X_{m}\left(i\right)$ and $X_{m+1}\left(i\right)$ can be defined respectively as:

(2) $$C_{i}^{m}\left(r\right)=\sum_{j=1,j\neq i}^{N-m}\Phi \left(m,i,j,r\right)$$

(3) $$C_{i}^{m+1}\left(r\right)=\sum_{j=1,j\neq i}^{N-m}\Phi \left(m+1,i,j,r\right)$$

where Φ(m,i,j,r) is defined as:

(4) $$\Phi \left(m,i,j,r\right)=\{\begin{array}{c} 1,\;\;\;d\left(m,i,j\right)\leq r\\ 0,\;\;\;\;\;\;\;\mathrm{otherwise} \end{array}\;$$

Then the probability that two vectors of length *m* will match is defined as:

(5) $$B^{m}\left(r\right)=\frac{1}{N-m}\sum_{i=1}^{N-m}\frac{1}{N-m-1}C_{i}^{m}\left(r\right)$$

and the probability that two vectors of length *m*+1 will match as:

(6) $$A^{m}\left(r\right)=\frac{1}{N-m}\sum_{i=1}^{N-m}\frac{1}{N-m-1}C_{i}^{m+1}\left(r\right)$$

Finally, we define the sample entropy as:

(7) $$SampEn\left(m,r,N\right)=-ln\frac{A^{m}\left(r\right)}{B^{m}\left(r\right)}$$

**Figure A1:**
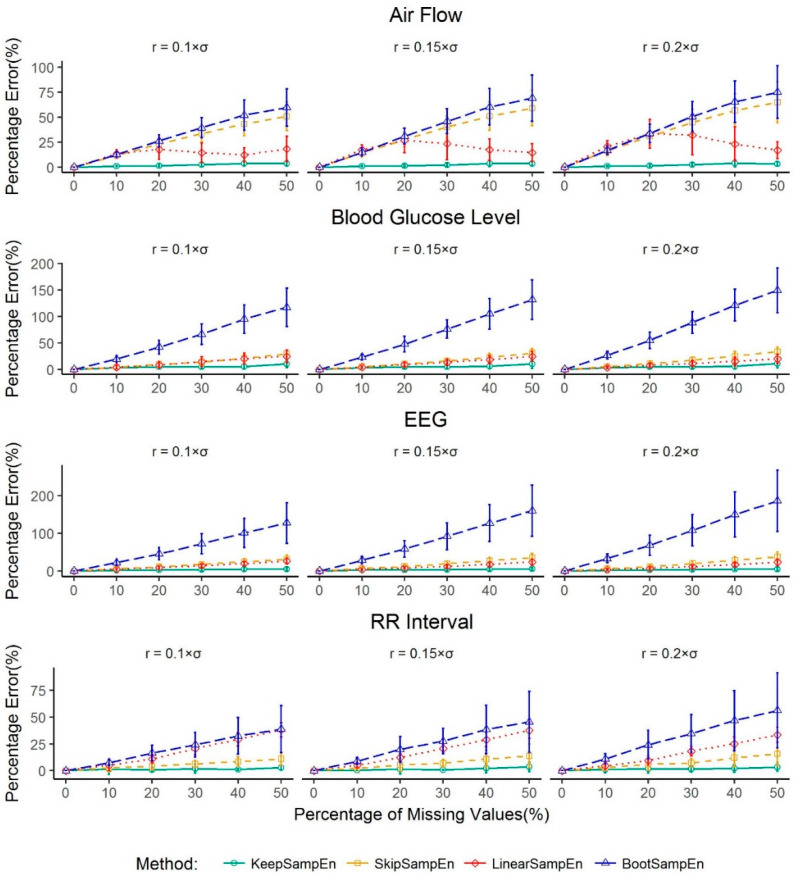
Percentage errors for each method in three types of continuously monitoring physiological signals: blood glucose level (top panel), RR interval (middle panel), air flow (bottom panel). Each panel includes three conditions: *r* = 0.1 × *σ* (**left**), *r* = 0.15 × *σ* (**middle**) and *r* = 0.2 × *σ* (**right**). Values are given as means ± standard deviation. The results for BootSampEn are not shown in the Figure 2 when the percentage error is too large and out of range of the figure.

Pincus [22] has proved that when the parameter *m* = 2 and *r* is between 0.1 × *σ* and 0.25 × *σ*, the sample entropy can retain enough information from time series and obtain effective statistical properties. Different *r* can give different conditional probability estimates. We have showed that there is no percentage error difference of four methods in four types data when *r* equal to 0.1 × *σ*, 0.15 × *σ* and 0.2 × *σ* (Appendix A, Figure A1). For different *r*, the trend of percentage error fluctuating with the percentage of missing values is consistent, so the parameters we used are: *m* = 2 and *r* = 0.15 × *σ*, where σ is the standard deviation of a time series.

### 2.2. KeepSampEn, SkipSampEn, LinearSampEn and BootSampEn

The definition of sample entropy cannot be directly used on time series data containing missing values. In order to solve this problem, a common method [16,23] is to remove the missing values and connect the remaining points into a single time series, which we denote as SkipSampEn. Additionally, interpolation is commonly used in handling missing values [24]. Two interpolation methods, linear interpolation and bootstrapping [25,26] which are denoted as LinearSampEn and BootSampEn respectively, are also addressed in our article. For the bootstrapping method, as described in Keun’s articles [25,26], ten reconstruction time series are generated from each time series containing missing values using bootstrapping method and the average value of entropy is obtained.

The major feature of our method, KeepSampEn, is that a new screening condition is added when $A^{m}\left(r\right)$ and $B^{m}\left(r\right)$ are calculated, which is that both $X_{m+1}\left(i\right)$ and $X_{m+1}\left(j\right)$ must contain only non-missing values. This condition not only ensures the existence of the distance function $d\left[X_{m+1}\left(i\right),X_{m+1}\left(j\right)\right]$, but also excludes the unmatched situation that $d\left[X_{m}\left(i\right),X_{m}\left(j\right)\right]$ exists but $d\left[X_{m+1}\left(i\right),X_{m+1}\left(j\right)\right]$ does not exist. So KeepSampEn is still the negative natural logarithm of the conditional probability $\frac{A^{m}\left(r\right)}{B^{m}\left(r\right)}$, but excludes the number of vector pairs of length *m* and *m* + 1 which contain missing values. This reduces the impact of missing values on the calculated value of sample entropy. In addition, in KeepSampEn, the standard deviation σ used to determine the tolerance value *r* (*r* = 0.15 × *σ*) is computed using only non-missing values, thus eliminating the impact of imputed values on the tolerance value. KeepSampEn is implemented in C and extended to multiscale sample entropy on the basis of mse.c from Costa et al. [20,27], other methods and the overall analysis are implemented in R language.

### 2.3. Experimental Datasets 

To evaluate the utility of our methods compared with existing methods, some common types of physiological signals are investigated. Physiological signal is divided into electrical signals and non-electrical signals. Electrical signals mainly include electrocardiogram (ECG), electroencephalogram (EEG) and electromyogram (EMG) signals. Sample entropy of electrical signals can be used to measure the complexity of the nervous system (ECG corresponds to cardiac nervous system, EEG corresponds to central nervous system, EMG corresponds to motor nerves). Since the purpose of measurement (complexity of nervous system) and signal type (electrical signals) are consistent, we selected the ECG and EEG signal as a representative for analysis and speculated that our method can obtain the similar results in EMG data too. In the non-electrical signal, we chose airflow data and blood glucose data for analysis. They can measure the complexity of the respiratory and glycemic metabolic systems respectively which correspond to respiratory diseases, diabetes and complications. Due to the high cost and low representativeness, other non-electrical signals weren’t analyzed. In summary, these four types of data contain most of the physiological signals that can be used for continuous measurements, which can analyze the complexity of cardiovascular, diabetes, respiratory diseases and their complications. 

For respiratory data, we used the retrospective data in a published study [28] for one regularly treated, chronic obstructive pulmonary disease patient in Beijing Chaoyang Hospital. Specifically we used the air flow data collected from ventilators over the course of 20 nights. In one night, the 5 Hz ventilator collects around 100,000 total points. However due to common issues in data collection, some measuring points are inevitably missing. Therefore, certain sections of respiratory sequences without missing points were selected for the analysis in this paper. All glucose data were obtained from the First Affiliated Hospital of Guangzhou Medical University. Twenty patients with type 2 diabetes (10 males and 10 females) used the Glutalor (Glutalor Medical Inc, Exton, PA, USA) for blood glucose measurement. That study was conducted according to the principles of the Helsinki Declaration and all participants gave their informed consent. The device measured blood glucose automatically every three minutes over the 7-day metrical period. The ECG data were obtained from the Fantasia Database in the PhysioNet (https://physionet.org/physiobank/database/fantasia/). Twenty young healthy subjects (21–34 years old) were selected and the original ECG sampling frequency was 250 Hz. They were measured while the study subjects were in a resting state while they were lying down. The RR interval is a time interval where two consecutive R waves crest in the ECG, and the sample entropy of it can reflect the complexity of ECG. After conversion, we obtained 20 original data series made up of RR intervals. The number of male and female subjects were equal. The EEG data were obtained from the CHB-MIT Scalp Database in the PhysioNet (https://www.physionet.org/pn6/chbmit/). Twenty pediatric epilepsy patients (1.5–22 years old) were selected and the original EEG sampling frequency was 256 Hz. We used the first column of each EEG data for analysis. Yentes et al. [29] found that sample entropy values stabilize around a length of 2000 points. Thus, except to verify the robustness to the impact of data size, we analyze the first 4000 points of each signals to rule out the effect of data length on the results (the length of glucose is only 2500, which is the maximum size in the dataset.). The choice of 4000 continuous points can make the shortest time series to reach 2000 points when the missing percentage reaches the maximum we set (i.e., 50%). 

### 2.4. Scheme for Generating Missing Values

In actual practice, many situations lead to missing values. We consider two common situations: a single missing point and a group of missing points at a time. An example of the first situation is that outliers [20,30] must be identified and excluded in heartbeat signals before calculating sample entropy. A common example of the second situation is a disruption in the wireless or network transmission of data which may cause a group of missing values [31,32]. 

**Figure 1:**
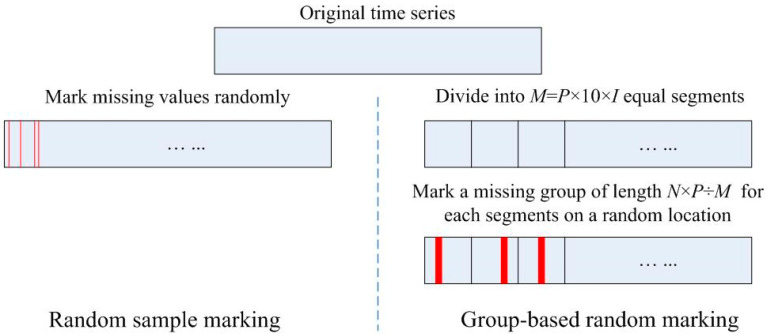
Two schemes for generating missing values.

Given a continuous time series having *N* points without missing values, we calculate its sample entropy. For convenience, we denote it as original entropy value. The total number of points marked as missing values is determined by the percentage of marked missing points *P*. The *P* values adopted in our research are 0% (baseline), 10%, 20%, 30%, 40%, 50%, respectively. We have designed two schemes to simulate the distribution of missing values: random sample marking and group-based random marking (Figure 1), which correspond to the two common situations that create missing values in time series data respectively.

Random sample marking: In this scheme, the missing points are randomly marked in the time series. First, the count of missing values to be marked *C* is calculated in accordance with the specified missing percentage *P* multiplied by the total number *N* (*C* = *P* × *N*). Then, *C* positions are selected randomly from the original time series without replacement. Finally, each value on these positions is replaced by a special identifier (*NA*, for example, is commonly used in R) to indicate the missing values.

Group-based random marking: In this scheme, continuous missing points are seen as a group and the starting position of the group is randomly chosen. The process is displayed in Figure 1. First, the original time series is divided into *M* segments on average, where *M* (*M* = *P* × 10 × *I*, *I* = 1, 2, 3...) is determined by a tunable parameter factor *I*. Second, we mark a missing group of length *N*×*P*÷*M* for each segment, and the starting position of each group is selected randomly in the segment.

By marking some points as missing values, a time series containing missing values is generated. Then we calculate the entropy values of the generated data using the various methods and compare them with the original entropy value. The performance of a method is evaluated using percentage error defined as follows:

$$percentage\;error=\left|\frac{x-x_{0}}{x_{0}}\right|\times 100\%$$

where *x* is the entropy value of the modified time series with missing values; $x_{0}$ is the entropy value of the original time series without missing values.

The percentage error represents the percentage of absolute deviation of the experimental entropy value with missing values from the original entropy value based on the original dataset. Thus, it can be used to evaluate the performance of a method in handling missing values. The smaller the percentage error, the better the method.

## 3. Results

### 3.1. Robustness to the Impact of Physiological Types

**Figure 2:**
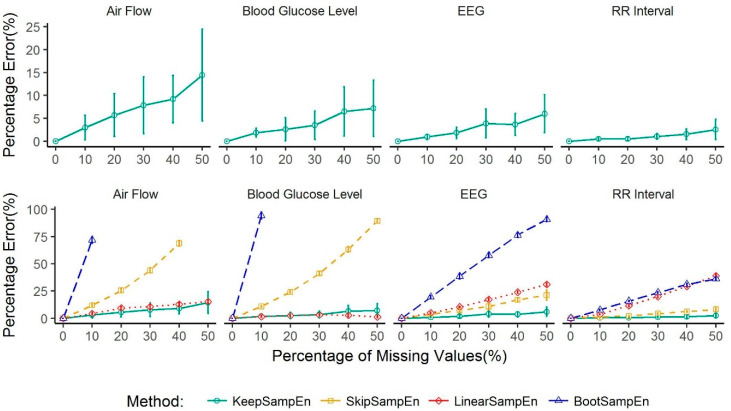
Performance of methods for handling missing values in four types of continuously monitoring physiological signals: air flow (**left**), blood glucose level (middle left-skewed), EEG (middle right-skewed), RR interval (**right**). Values are given as means ± standard deviation. The results for BootSampEn are not shown in the Figure 2 when the percentage error is too large and out of range of the figure.

To investigate whether our new KeepSampEn method is applicable to various types of physiological signals, we applied our method to four common types of physiological signals: air flow data, RR interval data (from ECG), EEG data and blood glucose levels. We first randomly selected one signal from each type of datasets, then used the random sample marking scheme to generate test data, finally repeated each method 10 times for a given proportion of missing values. The performance as measured by percentage error is shown in the top panels of Figure 2. The results clearly show that the KeepSampEn method had an average percentage error lower than 15% even in the situation with 50% of the values missing (top panels in Figure 2) in each of the four types of physiological signals. This demonstrates how our method has good performance and applicability over most common types of physiological signals. 

Next, the performance of KeepSampEn is compared to the three other methods: SkipSampEn, LinearSampEn and BootSampEn. The results are shown in the bottom panels of Figure 2. KeepSampEn achieved the lowest percentage error in each percentage of missing values for each type of data except for blood glucose levels missing a high percentage (more than 40%) of values. 

**Figure 3:**
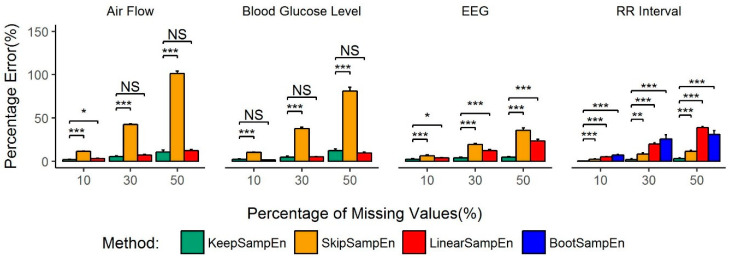
Average percentage errors for each method in four types of continuously monitoring physiological signals: air flow (**left**), blood glucose level (middle left-skewed), RR interval (**right**). The percentage errors by BootSampEn in the left and middle panels are higher than 120% and are not shown in the figure. Values are given as means ± standard error. NS means *p* > 0.05, * means *p* < 0.05, ** means *p* < 0.01, *** means *p* < 0.001.

To verify the stability of the result, we selected randomly 20 records from each dataset, then used the random sample marking scheme to generate test data, finally calculated percentage errors and make pairwise comparison by paired *T*-test. The results are shown in Figure 3, which is same as Figure 2. In this scenario where a high percentage of values for blood glucose levels are missing, the percentage error of KeepSampEn is only slightly higher than LinearSampEn without any significant difference (*p*-value > 0.05). 

BootSampEn performed poorly for all the four types of data. SkipSampEn has a good performance for RR interval data but a poor performance for rest three types of data. LinearSampEn has a good performance for air flow and blood glucose level but a poor performance for RR interval data and EEG data. By contrast, the performance of the new KeepSampEn method was robustly low for all four types of data (Figure 2 and Figure 3). This demonstrates how our method is robust to data type. In addition, the percentage error increased rapidly when the percentage of missing values increased for BootSampEn for all four types of data, for SkipSampEn in air flow and blood glucose levels, and for LinearSampEn in RR interval data and EEG data (Bottom panels of Figure 2). By contrast, our method did not demonstrate such sensitivity for any of the four types of data considered, which indicates another aspect of how robust the method is.

### 3.2. Robustness to the Impact of Data Size

**Figure 4:**
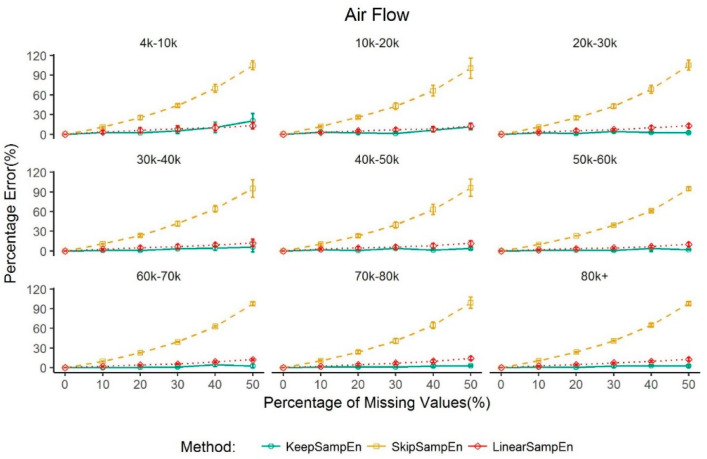
Performance of methods for handling missing values in air flow time series of nine large data sizes. Values are given as mean ± standard deviation. The percentage error for BootSampEn is out of the range and not shown in the figure.

To investigate whether the KeepSampEn method is robust to data size (i.e., the length of the time series in a study), we first applied our method to nine datasets with a large size (4k–10k, 10k–20k, 20k–30k, 30k–40k, 40k–50k, 50k–60k, 60k–70k, 70k–80k, 80k+, where 1k represents 1000 data points) from the air flow dataset. For each data size, we selected three time series randomly for analysis. The performance is shown Figure 4. The result shows that, among the four methods of handling missing values, our method KeepSampEn obtains the smallest percentage error rate almost in any data size. The result further shows that KeepSampEn can effectively control the percentage error below 4.53% regardless of the amount of data when the percentage of missing value is less than 30%. When the percentage of missing value is above 30% but less than 50%, the percentage error can be greater than 5% but within 15%. 

Considering the need of calculating sample entropy on a dataset with a small size in real clinical settings, we further explore the case of a dataset with a small size (i.e., 0.5k, 1k, 2k) from four types of physiological signals (i.e., airflow, glucose level, EEG and RR interval). Again, our proposed method achieves the smallest percentage error among the four methods in nearly all the settings except for airflow.

**Figure 5:**
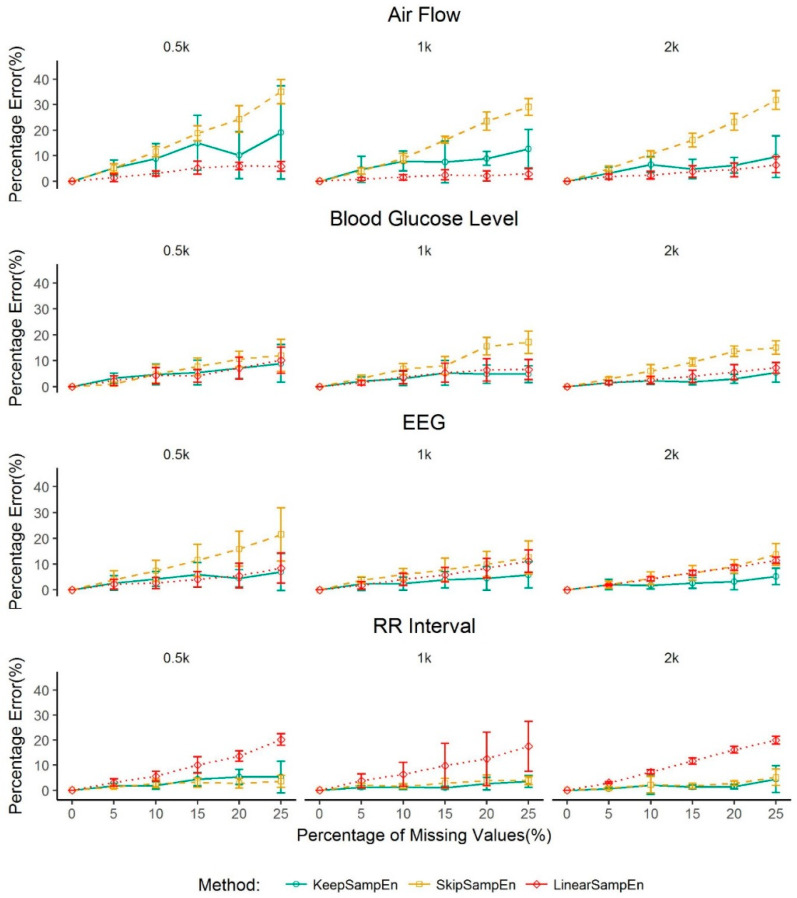
Performance of methods for handling missing values in a dataset with a small size (i.e., less than 2000 data points) for four types of physiological signals (i.e., air flow, blood glucose level, EEG and RR interval. Values are given as mean ± standard deviation. The percentage error for BootSampEn is out of the range and not shown in the figure.

Based on the performance shown in Figure 5, our method can control the percentage error to be less than 5% for a small dataset with a small percentage (i.e., lower than 15%) of missing values for the glucose, RR interval and EEG data. For air flow data, linearSampEn performs better than our method for a small dataset. However, our method controls the percentage error to be less than 4.63% when percentage of missing value is 5% or less. 

### 3.3. Robustness to the Impact of Schemes for Generating Missing Values

**Figure 6:**
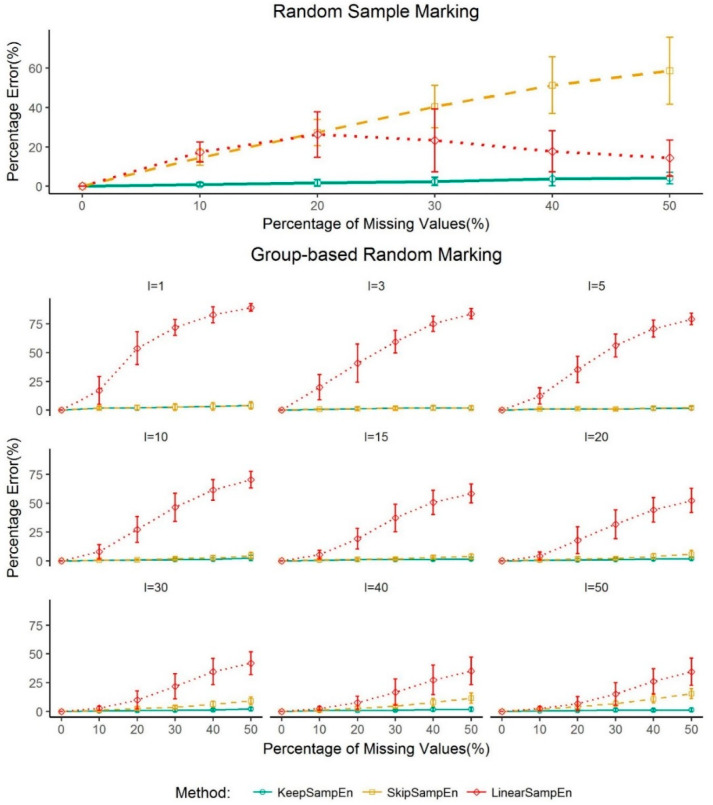
Percentage errors for each method using random sample marking and group-based random marking schemes to generate missing values, respectively. Values are given as mean±standard deviation. The percentage error for BootSampEn is out of the range and not shown in the figure.

To investigate whether our method is robust to the impact of schemes for generating missing values, we explore random sample marking and group-based random marking schemes with Factor *I* = (1, 3, 5, 10, 15, 20, 30, 40, 50) as described in the Method Section. Note, the factor *I* indicates the degree of scatterings of missing values. The larger the value of *I*, the more scattered the missing values. We selected randomly 20 signals from the air flow dataset, used two kinds of scheme to generate test data, then calculated the average percentage error for each proportion of missing values. The performance is shown Figure 6. The percentage error of the proposed KeepSampEn method remains low for both schemes, and it is low regardless of the scatter of the missing values (Figure 6). Thus, the new method is robust when it comes to how the missing values are generated. 

It worth noting that LinearSampEn achieved a low percentage error, similar to KeepSampEn, when the missing values were generated randomly. However, it has a much higher percentage errors when the missing values are grouped together (red lines in Figure 6).

### 3.4. Exploration of the Computational Complexity of KeepSampEn

**Figure 7:**
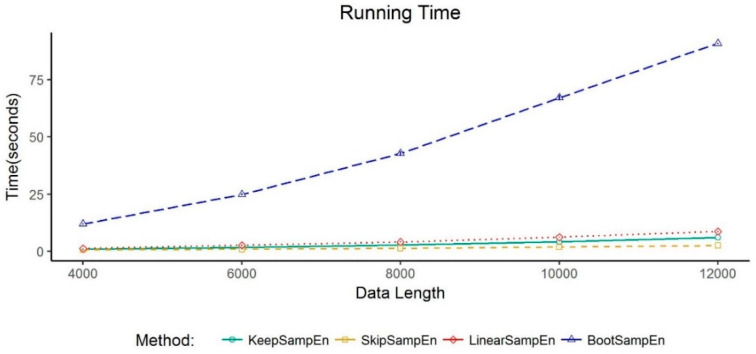
Evaluation of the running time of four methods for handling missing values. Each method is run on five values of percentage of missing values (i.e., 10%, 20%, 30%, 40% and 50%) on the air flow dataset. The running time for them are summed up to be the total running time. This process is repeated 10 times. The average total running time for the 10 repeats is shown in the *y*-axis. Values are given as mean ± standard deviation. Note, the standard deviation is tiny so that it can hardly been seen in the figure.

We evaluate the computational complexity by calculating running time of four methods on the data with the same length (time complexity). The time for calculating sample entropy based on the time series without any missing value has O(n^2^). For convenience, we denote this running time as the base so that the running time of all the four methods for handling missing values can be compared with it. SkipSampEn takes slightly less time than the base due to the reduction of the number of points. KeepSampEn takes almost the same time as the base. LinearSampEn takes slightly more time than the base due to additional interpolation operations. BootSampEn takes 10 times as much time as the base because it includes resampling and averaging 10 times. We further use a list of air flow data for verification. The result shown in Figure 7 confirms the theoretical derivation. 

## 4. Discussion

Sample entropy is a widely used metric for assessing the irregularity of physiological signals. In practice, missing values or outliers commonly exist in the data of physiological signals. If we use conventional methods (neglect/interpolation) to calculate the signal with missing values, the resulting value will have some error from the original series. In this paper, we propose a new method, KeepSampEn, to minimize the error due to missing values in sample entropy calculation. This approach is less intrusive to the structure of the original data and provides a theoretical advantage in handling missing values in the analysis of sample entropy. 

We further studied the utility, applicability and robustness of our method in practice by designing common different datasets with missing values from experimental data without missing values. This was done for most of types of physiological signals and corresponding diseases. In this paper, we use percentage error to judge the quality of various missing value processing methods. The results of our analysis demonstrate that for several common physiological data, our method can minimize the influence of missing values in the sample entropy calculation. In addition, the performance of our method is stable and robust, whereas other methods have either a poor or an unstable performance (Figure 3). 

One limitation for the use of our method is data type. We only verified that our method is suitable for ECG data, EEG data, blood glucose data, airflow data rather than all physiological data. For other electrical physiological signals such as EMG signals, we speculate that conclusion is consistent with ECG and EEG signals due to the same signal types and measurement purposes. However, if researchers want to apply KeepSampEn to other types of data, simple verification is required.

We also investigated the performance of our method in various large sizes of data. The results show that the entropy value obtained using our method is always close to the true value (i.e., the entropy value from the original data without missing values), with a percentage error less than 4.53% when the percentage of missing values is less than 30% for data sizes varying from 4000 to 80,000. We further see that the percentage error can be greater than 5% but within 15% when the percentage of missing value is above 30% but less than 50%. For a dataset with a small size (i.e., from 500 to 2000 data points), our method can control the percentage error to be less than 5% for blood glucose level, EEG and RR interval when the percentage of missing values is less than 15%. This contrasts with the entropy values obtained using other methods which greatly deviated from the true value (Figure 4 and Figure 5). These results may provide valuable information for data quality control in practice. That is, for the physiological signals, when a dataset has a percentage of missing values less than 30% for a large data size or 15% for a small data size of total series due to equipment or operational errors, our method can rescue the data through minimizing the impact of missing value (i.e., controlling the percentage error of the sample entropy to be less than 5%). On the other hand, if the percentage of missing values reaches 30% or more for a large data size or 15% or more for a small data size, the result of our research indicates that the calculated value of sample entropy is not reliable anymore even if we adopt the best method for handling missing values. In such a case, the data may be screened out from further analysis on sample entropy. It should be mentioned that, for air flow data, linearSampEn performs better than our method for a small dataset. However, it is easy and convenient to obtain a large dataset for air flow in real clinical settings and the size of airflow datasets is usually large in the clinical setting. Thus, we should usually not worry about the case of a small dataset of air flow. Moreover, even for air flow data, our method controls the percentage error to be less than 4.63% when percentage of missing value is 5% or less. 

We further investigated whether our method will be affected by how the missing values are generated. The results indicate that our method is robust to how the missing values are generated in the continuous monitoring of physiological signals (Figure 6). 

In conclusion, our proposed KeepSampEn method has the following merits for handling missing values in the analysis of entropy for continuously monitored physiological data. First, unlike the usual ways by modifying the input data, our method keeps the input data unchanged and modifies the calculation process, which is less intrusive to the structure of original data. Second, it is effective and applicable to most common types of physiological signal data for a variety of diseases. Third, it is robust in not only how long the data is but also how the missing values are generated. This is a marked improvement over the currently used methods for handling missing values in analysis of sample entropy. With these merits, our proposed method should have broad utility in handling missing values in the analysis of sample entropy for continuously monitored physiological signals. 

## Supplementary Materials

The supplementary materials are available online at https://www.mdpi.com/1099-4300/21/3/274/s1.
