# Supplementary material for: An Improved Method of Handling Missing Values in the Analysis of Sample Entropy for Continuous Monitoring of Physiological Signals
Source: Entropy (Basel). 2019 Mar 12;21(3):274. doi: 10.3390/e21030274 (PMC7514754; doi:10.3390/e21030274)
Supplement: Supplementary file 1 [file entropy-21-00274-s001.zip › supplementary/supplementaryMaterial.doc]

Supplementary Material: How to Compile and Run KeepSampEn in R

Xinzheng Dong, Chang Chen, Qingshan Geng, Zhixin Cao, Xiaoyan Chen, Jinxiang Lin, Yu Jin, Zhaozhi Zhang, Yan Shi, Joseph F. Heyse and Xiaohua Douglas Zhang

Introduction

The source codes for SkipSampEn is based on mse.c which can be downloaded from <https://www.physionet.org/physiotools/mse/mse.c>. We modified the data input part of the program to calculate the entropy of the time series with missing values. For reader's convenience of reading, we remove the part of mse.c that contains multi-scale entropy and simplify it. We call it: SampEn_skipNA.c. The major codes for calculating KeepSampEn are implemented in the C source file, SampEn_keepNA.c. It is also the improvement based on mse.c. We add the process of dealing with missing data.

The result format of the R demo is as follows:

...

proportion: proportion of missing values

keep: entropy calculated by the KeepSampEn method

skip: entropy calculated by the SkipSampEn method

keep_error: percentage error of the KeepSampEn method (%)

skip_error: percentage error of the SkipSampEn method (%)

Preparation (What you need to do)

1. The RR interval data can be download from <https://physionet.org/cgi-bin/atm/ATM>, researchers can choose the database: Fantasia Database(fantasia) and Toolbox: Show RR intervals as text, then they can download the output file: rr.txt.

Notes: Other data can be download from http://quantitativelabfhsdev.pclan.umac.mo/analytic-tool/

2. Create a local folder “heartrate data” to store a series of data. Put the folder into the working directory. Actually, all input files should be copied to the working directory.
3. The SampEn_keepNA.c and SampEn_skipNA.c should be copied to the working directory (the default is the same directory as the R script file).
4. Open a new R script and copy the demo R script content to it.

Main steps of the demo R script (What R demo does)

1. Load the original data of length 4000 from the input file "heartrate data/rr.txt".
2. Generate a group of data containing missing values (proportion:0,10%,20%,30%,40%,50%) from the original data using random sample marking.
3. Run KeepSampEn(SampEn_keepNA.c) and SkipSampEn(SampEn_skipNA.c) methods to calculate entropy for each proportion of missing values.
4. Repeat step2 - step3 10 times.
5. Calculate percentage errors of both methods.
6. Print the result.

Demo R script

#begin of demo script

**#A function of compiling and running C codes in R**

run.exe <- function(m){

shell( paste0("gcc -o", m, " -O ", m, ".c -lm -w"), wait=TRUE, intern=FALSE, invisible=TRUE )

shell( paste0(m,".exe -I ", length(index), " -F filelist.txt > ", m,".output"), wait=TRUE, intern=FALSE, invisible=TRUE)

}

**#Global configuration**

datapath<-"timeseries.txt"

factors<-c(0:5)/10

index<-c(1:4000)

\#**Get result data from the output file of specified method**

get.result<-function(m){

input<-file(paste0(m,".output"))

text=readLines(input)

header <- ""

for(f in factors)

{

header <- paste0(header, formatC(f, digits = 4, format = "f"), '\t')

}

position <- grep(header, text)

h <-as.numeric(strsplit(header, "\t")[[1]])

proportion<-h

df=as.data.frame(proportion)

position <- position+1

y<-strsplit(text[position], "\t")[[1]]

y<-as.numeric(y)

df<-cbind(df, y)

close(input)

colnames(df)<-c("proportion", as.character(1:1))

df

}

**#Generate file with missing values of a proportion**

random.gen.file<-function(data, outname, factor){

missing <- c()

if(nrow(data)>=length(index))

{

sample_size <- floor(factor*length(index))

missing <- sample(index,sample_size)

}

else

{

sample_size <- floor(factor*nrow(data))

missing <- sample(1:nrow(data), sample_size)

}

data[missing,] <- NA

\# print(sum(complete.cases(data)))

#matrixplot(data)

write.table(data, file = outname, row.names = F, col.names = F, quote = F)

}

**#Generate files with missing values (0%-50%)**

gen.missing<-function(type="random"){

data = read.csv(datapath, header=FALSE)

sink("filelist.txt")

for(f in factors){

outname=sprintf("%f_%s", f, datapath)

cat(outname)

cat("\n")

switch(type,

random = random.gen.file(data, outname, f),

warning("wrong type for gen.missing!"))

\# break

}

sink()

}

**#Main steps of the demo R script**

repeat.random.method <- function(type)

{

\# select the test segment

selected.data <- c()

switch(type,

heart_rate = {

data <- read.csv("heartrate data/rr.txt",header=T,sep="\t", nrows = 4000)

selected.data <- data[,3]

},

warning("wrong type for repeat.random.method!"))

write.table(selected.data, file = datapath, row.names = F, col.names = F, quote = F)

dfall <- data.frame()

loopcount <- 10

for(i in 1:loopcount)

{

gen.missing()

run.exe("SampEn_keepNA")

run.exe("SampEn_skipNA")

df1 <- get.result("SampEn_keepNA")

df2 <- get.result("SampEn_skipNA")

df<-cbind(df1, df2[-1])

colnames(df)<-c("proportion", "keep", "skip")

dfall <- rbind(dfall,df)

}

base <- dfall[dfall$proportion==0, "keep"]

basec <- rep(base, each=6)

dfall <- cbind(dfall, keep_error=abs(dfall$keep-basec)*100/basec)

dfall <- cbind(dfall, skip_error=abs(dfall$skip-basec)*100/basec)

dfall$proportion <- dfall$proportion*100

print(dfall)

}

repeat.random.method("heart_rate")

#end of demo script
